# Hatred out of love or love can be all-inclusive? Moderating effects of employee status and organizational affective commitment on the relationship between turnover intention and CWB

**DOI:** 10.3389/fpsyg.2022.993169

**Published:** 2023-01-04

**Authors:** Xiaolang Liu, Wenzhu Lu, Shanshi Liu, Chuanyan Qin

**Affiliations:** ^1^School of Management, Guangdong University of Technology, Guangzhou, Guangdong, China; ^2^School of Business Administration, South China University of Technology, Guangzhou, China; ^3^School of Medical Business, Guangdong Pharmaceutical University, Guangzhou, Guangdong, China

**Keywords:** turnover intention, employment type, organizational affective commitment, CWB, hybrid employment

## Abstract

Owing to the prevalence of flexible employment practices around the world and increasingly loose employee-organization relationships, employee turnover intention is gradually becoming normalized. This study aimed to examine the counterproductive work behaviors (CWB) of employees with turnover intention in the hybrid employment context. Drawing on the psychological contract process perspective, this research endeavored to examine whether higher turnover intention is associated with greater levels of CWB and to determine whether and how the association between turnover intention and CWB differs across temporary and permanent workers by considering organizational affective commitment. The results of analyzing 211 pairs of two-wave subordinate–supervisor matching data from a Chinese service company indicated that turnover intention is positively related to CWB, and the association is stronger for temporary workers than permanent ones. Such difference is caused by permanent workers’ higher organizational affective commitment than temporary workers. The findings’ implications for theory and research are provided in hybrid employment.

## 1. Introduction

Turnover is considered costly for organizations because it involves replacing a worker who has left; this has attracted considerable attention from academics and practitioners (Jiang et al., 2019; Richard et al., 2020; Laulie and Morgeson, 2021). Several studies have focused on the factors predicting employee turnovers, such as organizational factors (e.g., identity strain, customer incivility, and diversity; Stewart, 2011; Chris et al., 2022; Mistry et al., 2022; Rafiq et al., 2022) and individual factors (e.g., age and personality; De Meulenaere et al., 2022). Furthermore, prior research has shown that there is a significant difference between an employee’s turnover intention and the actual exit (Cohen et al., 2016; Mai et al., 2016). Therefore, it is imperative for scholars to further investigate the influence of employee turnover intention on employees’ subsequent behaviors while they are still in the organization.

While a few studies have provided evidence linking turnover intentions with discretionary behaviors at work (i.e., organizational citizenship behaviors, voice behavior; Burris et al., 2008; Mai et al., 2016; Verbruggen and Van Emmerik, 2020), these studies were cross-sectional or homologous (Lee et al., 2021). Furthermore, the aforementioned studies were conducted in the traditional employment context (Christian and Ellis, 2014; Mai et al., 2016), without taking into account the new employment environment. Accordingly, the first objective of the present study is to investigate how employees with turnover intention engage in counterproductive work behaviors (CWB) while continuing to remain with the organization by employing multi-source and multi-phase data. In this context, employees’ CWB refers to employees’ voluntary behaviors, such as violating organizational norms and threatening the well-being of the organization and/or its members (Bennett and Robinson, 2000; Wu et al., 2022). We focus on the impact of turnover intention on CWB because such behaviors from employees can have a more devastating impact on organizations and individuals than other behaviors (Greco et al., 2015).

Furthermore, organizations struggle to implement leaner cost structures and obtain competitive strength *via* alternative work arrangements, such as the use of temporary employees (Cappelli and Keller, 2013; De Meulenaere et al., 2022). Numerous scholars have noted differences in the employment status and psychological contract content among temporary and permanent employees. In particular, prior studies have suggested that temporary employees possess less job autonomy, lower team commitment, and reduced intrinsic motivation than permanent employees (Boyce et al., 2007; de Jong et al., 2019). Although references to differences in the psychological contract content between temporary and permanent employees are pervasive in the literature (De Cuyper et al., 2009), research has yet to explore whether permanent and temporary employees have different reactions to turnover intention and how these effects occur. Therefore, the second aim of this study is to examine whether and how employee turnover intention can have a distinct impact on employees’ CWB across permanent and temporary workers.

**Figure 1:**
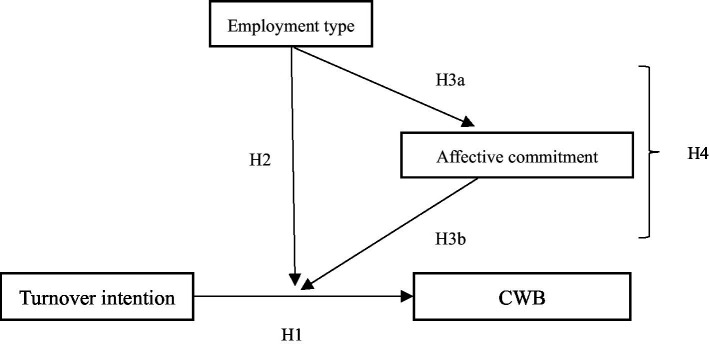
The proposed model.

To address these important research gaps, we draw upon the psychological contract processes perspective (Dulac et al., 2008; Rousseau et al., 2018) to explore whether employee turnover intention leads to CWB and to determine the differences between temporary and permanent workers within this association. The proposed model is depicted in Figure 1. The psychological contract theory has emerged as a tool for comprehending the complex relationships between employees and organizations (Dabos and Rousseau, 2004; Cullinane and Dundon, 2006), thus impacting employee performance and loyalty (Bal et al., 2013; Bordia et al., 2017). The psychological contract represents an employee’s perception of a reciprocal obligation between themselves and the organization (Rousseau et al., 2018), which contains transactional and relational components; employees can orient to one or both components. When an employee resolves to leave the organization, it usually means the breakdown of this reciprocal relationship, likely triggering the employee’s CWB.

Moreover, we propose that the relationship between turnover intention and CWB is complex across changes in the psychological contract. In essence, the transition from the intention to leave to the actual resignation is a process of continuous decision-making by employees, and the type of contract between the employee and the organization plays a key role during this process. The dissolution of the relational contract and that of the transactional contract have inconsistent effects on employees. We first applied employment type as a representation of the type of psychological contract. However, studies have shown that temporary workers may also exhibit a commitment to the organization or identify their organization (Breugel et al., 2005; Lapalme et al., 2011), thereby forming a complicated psychological contract. Therefore, we have chosen organizational effective commitment as the expression of the relational psychological contract (as shown in Table 1). We want to further confirm whether and how the difference between turnover intention and CWB among different employment types depends on their actual relationship status.

**Table 1:** Means, standard deviations, correlations of variables.

	Contract workers		Permanent workers													
	M	SD	M	SD	1	2	3	4	5	6
1. Gender	1.550	0.500	1.610	0.491						
2. Age	1.440	0.615	1.940	0.768	−0.116					
3. Education	2.790	0.934	3.210	0.759	0.310^***^	−0.012				
4. Tenure	2.870	1.233	4.300	1.159	0.094	0.501^***^	0.099			
5. TI	2.550	0.848	2.141	0.850	−0.016	−0.165^*^	−0.033	−0.141^*^		
6. AC	3.217	0.643	3.521	0.593	0.075	0.087	0.167^*^	0.142^*^	−0.400^***^	
7. CWB	2.738	0.520	2.306	0.480	0.038	−0.147	−0.131	−0.211^**^	0.596^***^	−0.399^***^

N (temporary workers) = 102; N (permanent workers) = 109. Gender coded: 1 = male, 0 = female. TI: turnover intention; AC: organizational affective commitment. Reliabilities are listed in parentheses.

*p < 0.05, **p < 0.01, ***p < 0.001 (two tailed).

The current research makes three important contributions to the literature. First, previous works on turnover have focused on the predictors of employees leaving, using turnover intention as the criterion (Hom et al., 2012). However, we considered the behavioral performance of employees with the intent to leave, adding a new perspective to the turnover literature.

Second, we clarified the relationship between turnover intention and CWB in flexible employment by considering temporary workers’ turnover intention, which contributes to the literature on turnover intention and the psychological contract. In particular, we explored whether and how temporary and permanent workers respond differently to the turnover intention with regard to CWB. Thus, our research also supplements previous studies on the differences in the behaviors of employees with different statuses from the perspective of the psychological contract theory.

Third, by drawing upon the psychological contract process perspective (Dulac et al., 2008; Rousseau et al., 2018), we pay more attention to the dynamic model of psychological contract research. From the psychological contract process perspective, studying employees’ turnover intention (psychological contract breakdown) and the complex relationship between relationship types and relationship bases is of great significance for understanding the impact of employee–organization relationship formation, and termination in the Chinese context.

## 2. Hypothesis development

### 2.1. Turnover intention and CWB

CWB describes employees’ volitional behavior that potentially violates the legitimate interests of or does harm to an organization or its stakeholders (Sackett and DeVore, 2002). We propose that employees who consider leaving the current organization may engage in CWB to retaliate against the organization. According to the psychological contract theory, a mutually beneficial exchange relationship exists between an organization and its employees (Tsui et al., 2017). However, if an organization fails to fulfill its obligations, its employees’ motivation will be negatively impacted, and the psychological contract with the organization will be breached. Therefore, they will be more likely to reduce their obligations as a form of revenge (Christian and Ellis, 2014). Turnover intention is strongly predicted by psychological contract breaches in the relationship between individuals and an organization (Bravo et al., 2019). Prior research has indicated that turnover intention dampens employees’ relational contracts (Burris et al., 2008; Bravo et al., 2019). Once an employee decides to leave, their relational contract perception has changed; they are consciously disrupting the bonds of the contracts and do not consider what they owe their employer. This, in turn, results in employees distancing themselves from the organization’s relational components. Supporting our hypothesis, Burris et al. (2008) found that employees with high turnover intention are far less concerned about the benefits to the organization and exhibit psychological detachment from the organization (Burris et al., 2008). Thus, employees who intend to leave are no longer concerned about fulfilling their side of the exchange relationship by abiding by the organization’s rules and are more likely to engage in CWB.

Furthermore, employees are constrained by an organization’s formal rules and procedures. Employees complying with the rules are usually given rewards, such as promotion opportunities and higher salaries, and violating the rules is punished accordingly. However, once an employee decides to leave the organization, they are no longer dependent on the organization for income, a sense of belonging, or other basic needs. As such, employees with turnover intention are less likely to adhere to situational norms and more likely to behave unprofessionally (Middleton et al., 2018). Thus, employees are more likely to express their dissatisfaction with the organization and may thus choose to participate in CWB. Therefore, we propose the following hypothesis:

*Hypothesis 1*: Turnover intention is positively related to CWB.

To reveal the relative consequences of turnover intention on various groups of employees, we examine the moderating role of employment type (permanent vs. temporary). It is more difficult to make predictions about the relationship between turnover intention and CWBs for permanent workers compared to temporary workers.

On the one hand, due to the poor relationship basis with the organization, temporary employees are more likely to engage in CWB than permanent workers when they hold the turnover intention. According to the psychological contract theory, an employee’s employment status reflects the overall connection between the employee and the organization, and this can predict employee behaviors (Dulac et al., 2008; Rousseau et al., 2018). Temporary workers sign a contract with a predetermined expiration date and do not have an explicit agreement for long-term employment (Broschak and Davis-Blake, 2006). They are partially outside formal policies on integration and are usually treated as “plug-in” disposable resources (Broschak and Davis-Blake, 2006; De Stefano et al., 2019). An organizational practice for managing flexible workers is to invest in limited firm-specific human capital, such as lower payments, less training, and fewer promotion opportunities (Mooi-Reci and Wooden, 2017). This may enhance temporary workers’ perception of themselves as organizational outsiders and prevent their investment in an organization. There is empirical evidence that temporary workers perceive themselves to be “excluded from the organization” and feel “less a part of the organization” (De Cuyper et al., 2009). Thus, the basis of the employee–organization relationship is bounded by employment contracts. This forms a transactional contract between employees and organizations and indicates a low-quality exchange relationship. Due to poor employment relations with organizations, temporary workers do not expect long-term employment and do not exhibit much organizational involvement (de Ruyter et al., 2008).

Furthermore, the psychological contract theory also indicates that the psychological contract between an employee and an organization is rooted in the norm of reciprocity and the expectation that there will be a balance between obligations and entitlements on the organization’s part and the employee’s part (Conway and Briner, 2005; Shaw et al., 2009; Pan et al., 2020). Permanent employees usually exhibit high-quality entitlements and are more likely to perceive CWB as an inappropriate response because it may cause harm to another entity and weaken their connection with their organization. While temporary employees are expected to be more likely to engage in CWB as a way of expressing their dissatisfaction with an organization’s faults based on the negative reciprocity principle. Therefore, when temporary employees decide to leave an organization, CWB is more likely to serve as an indirect way of retaliating against the organization for discrimination, and they are more likely to seek retribution by engaging in CWB than permanent workers.

However, a case could also be made for a stronger relationship between turnover intention and CWB for permanent workers than for temporary workers. Research has consistently found that the experience of turnover intention leads to the perception that the employer has not fulfilled their obligations (Conway and Briner, 2005). Permanent employees are motivated to invest more resources and engagement in their organization due to their insider status than temporary employees with limited contact. Therefore, permanent employees with a high-quality relationship with the organization may be more sensitive to the perceived breach of the social exchange relationship that accompanies turnover intention (Probst et al., 2018a,b). Therefore, when permanent workers experience turnover intention, CWB is more likely to serve as an indirect way of retaliating against the organization for the perceived breach, and they are more likely to seek retribution by engaging in CWB than temporary employees. In contrast, the turnover intention is more acceptable and predictable for temporary workers because they sign a fixed contract with organizations. Consequently, temporary workers are less likely than permanent workers to opt for retaliation because they have anticipated their departure. Accordingly, we hypothesize the following:

*Hypothesis 2*: The relationship between turnover intention and CWB is moderated by employment type. In particular, the turnover intention is more/less likely to result in CWB for temporary employees than for permanent employees.

We also predict that employment type is related to organizational affective commitment. In particular, we suggest that permanent workers usually possess higher organizational affective commitment than temporary workers. Organizational affective commitment is described as the strength of an individual’s identification with and involvement in an organization; it includes (1) a strong belief in and acceptance of the organization’s goals and values; (2) a willingness to exert considerable effort on behalf of the organization; and (3) a strong desire to maintain their membership in the organization (Steers, 1977).

First, organizational affective commitment is related to job characteristics, work experience, and the employee–organization relationship, which includes role ambiguity, job satisfaction, perceived organizational support, employee empowerment, and organizational training (Steers, 1977; Neubert and Halbesleben, 2014; Hanaysha, 2016; Kim et al., 2016). We argue that employment type could be a predictor of organizational affective commitment. The psychological contract theory contends that a reciprocal exchange exists between an employee’s contribution and the organization’s inducement and that employees behave on the basis of the norm of reciprocity to seek a balance between the favorableness of their orientation toward the organization and the organization’s orientation toward them (Cropanzano et al., 2022). However, unlike permanent workers, temporary employees are usually associated with poor-quality jobs (Kalleberg, 2009). For example, several previous studies have shown that temporary employment is often associated with lower earnings, less access to health, and fewer pension benefits (Boyce et al., 2007; de Graaf-Zijl et al., 2009; Kalleberg, 2009). Moreover, employees on temporary contracts are typically assigned jobs involving routine, repetitive, and hazardous tasks, and an organization may be reluctant to invest in temporary workers’ training (Esteban-Pretel et al., 2011; Mooi-Reci and Wooden, 2017). Poor job conditions represent a low-quality exchange relationship between organizations and employees, which could cause temporary employees to be unwilling to invest considerable effort toward the organization’s goals. Therefore, we propose that temporary workers usually have lower levels of organizational affective commitment than permanent workers.

Furthermore, differentiated management of temporary workers causes them to perceive that the organization does not value their contributions. It signals to temporary employees that the organization is ignoring their socio-emotional needs, such as esteem, approval, and affiliation (Guillaume et al., 2019). Consequently, to balance the employee–organization relationship, temporary employees adjust their attitudes and behavior toward the organization. Discriminative management practices may cause temporary workers to perceive that they receive less organizational support and develop a deep sense of dissatisfaction, resulting in lower levels of organizational affective commitment. Therefore, temporary workers have lower organizational affective commitment than permanent workers. Based on this, we present the following hypothesis:

*Hypothesis 3a*: Employment type is related to organizational affective commitment. Temporary employees have a lower level of organizational affective commitment than permanent employees.

We also propose that organizational affective commitment being an outcome of employment type could help explain the association between turnover intention and CWB. We expect that high levels of organizational affective commitment can weaken or strengthen the positive relationship between turnover intention and CWB. Organizational literature has recognized that organizational affective commitment is a significant factor that determines employee behavior (Meyer et al., 2002). According to the psychological contract theory, employees with stronger affective commitment to their organization tend to behave in the organizations’ interests (de Jong et al., 2019). High-level organizational affective commitment binds individuals to organizations, which implies that employees are loyal to the organization and willing to exert considerable effort on its behalf (Bateman and Strasser, 1984). Committed employees may possess a sense of self and identity that are aligned with their organizations’ goals and values (Dawson et al., 2014). Thus, even if an employee’s current relationship with the organization is broken and they develop turnover intention, highly committed employees are less likely to seek retaliation due to their previous emotional connection with the organization. Furthermore, individuals with high organizational affective commitment are more likely to feel indebted to the organization and perform beyond general duties and responsibilities to achieve the organization’s goals (Dawson et al., 2014). Due to their duties and guilt toward the organization, employees may experience more psychological pressure when they engage in behaviors that harm the organization. Therefore, when high-commitment employees are willing to leave, they will make decisions consistent with organizational objectives and will not behave in a manner that harms the organization. Previous research has also provided empirical evidence for this argument; for instance, Meyer et al., (2002) suggested that employees with a high degree of commitment are less likely to engage in destructive behavior.

However, we also hypothesize that highly committed employees who develop turnover intention are more likely to react with CWB as compared to less committed employees. Employees with high affective commitment usually look forward to the organization’s realization of the relational psychological contract. Therefore, when such employees develop the intention to leave, they will be greatly impacted by the breakdown of this psychological contract. Employees who view their relationship with the organization favorably seek to reciprocate the costs and benefits received to maintain a balanced relationship. However, when such highly committed employees experience turnover intention, it creates an imbalanced relationship between them and the organization. The negative reciprocity principle indicates that individuals attempt to resolve an imbalanced relationship such that employees who are harmed by organizations retaliate by harming the organization. Therefore, we reason that, when highly committed employees experience turnover intention, they are more likely to retaliate with CWB to restore the balanced employee–organization relationship. Based on this, we present the following hypothesis:

*Hypothesis 3b*: Organizational affective commitment moderates the relationship between turnover intention and CWB. In particular, individuals with high organizational affective commitment would strengthen or weaken the positive relationship between turnover intention and CWB.

Regarding how temporary and permanent workers differ from each other in terms of the relationship between turnover intention and CWB, there are two competing predictions. One possible explanation for employees’ distinct responses to turnover intention is that employees’ organization–employee relationship foundation determines organizational commitment. As previously indicated, temporary employees usually possess lower organizational affective commitment than permanent ones. However, such an employee–organization relationship foundation could be a double-edged sword. On the one hand, organizational affective commitment generated by employment type lays the foundation for the employee–organization relationship, which indicates the connection with the organization and can mitigate the negative effect of turnover intention on CWB. Therefore, when the current employee–organization relationship breaks down accompanied by turnover intention, the original relationship foundation (i.e., organizational affective commitment) would prohibit permanent employees from harming the organization and enhance their ability to better cope with turnover intention. However, it is also possible that permanent employees possessing high-quality relationships with the organization may enable them to be more sensitive to the perceived breach of the psychological contract that accompanies turnover intention (Burris et al., 2008). In contrast, as temporary employees usually sign a contract with a predetermined expiration date and do not expect long-term employment (Katz and Krueger, 2019), they hold lower expectations of the organization and are thus less likely to retaliate against the organization by engaging in CWB.

In this study, we test the preceding two hypotheses, which propose that employment type influences employees’ organizational affective commitment and generate a competitive hypothesis regarding the moderating role of organizational affective commitment between turnover intention and CWB. Together, these two hypotheses predict that organizational affective commitment mediates the moderating effect of employment type on the relationship between turnover intention and CWB (Hypothesis 2). The type of mediated moderation that we expect is present when the employment type moderates the relationship between turnover intention and CWB, as in Hypothesis 1; the employment type influences organizational affective commitment, as in Hypothesis 3a; and organizational affective commitment moderates the relationship between turnover intention and CWB, as in Hypothesis 3b, thereby transmitting the moderating effect of the original moderator, employment type. Due to mediated moderation being present when a moderating effect is explained by a mediating process (e.g., Edwards and Lambert, 2007), our arguments give rise to the following hypothesis:

*Hypothesis 4*: Employment type moderates the effect of turnover intention on CWB, and this is mediated by organizational affective commitment.

## 3. Materials methods

### 3.1. Sample and procedures

In 2019, we collected data from a large service organization located in China that employed both contract workers and permanent workers. This service company’s business mainly involves airline service and civil aircraft maintenance. We mainly collected data from the company’s branches in Hainan, Changsha, and Xinjiang. In our surveys, a cover letter accompanying the questionnaire explained that the research would be used only for academic research and that we would not divulge the responses to anyone. At Time 1, the questionnaires were disseminated to 235 employees. The participants were asked to self-report their employment type, turnover intention, and organizational affective commitment. A total of 211 participants responded to the survey, yielding a response rate of 88.1%. At Time 2, 1 month later, we delivered the questionnaire to their immediate supervisors and requested them to rate their subordinates’ CWB. Based on the company’s personnel information, we matched the employees’ IDs with their supervisors’ evaluations. Of the respondents, 102 (48.3%) were temporary employees, and 119 (56.4%) were female.

### 3.2. Measures

#### 3.2.1. Turnover intention

Turnover intention was assessed with Aryee and Chay (2001) three-item scale. The participants responded using a five-point Likert scale, ranging from 1 (“strongly disagree”) to 5 (“strongly agree”). The example items are “I often think about quitting my job with my present organization” and “I will probably look for a new job within the next year.”

#### 3.2.2. Employment type

The employment type was divided into two: contract workers and temporary employees. It was measured by asking the respondents to answer which type applied to them.

#### 3.2.3. Organizational affective commitment

The organizational affective commitment was measured using Francesco and Chen (2004) six-item scale. The sample items include “I would be very happy to spend the rest of my career with this organization” and “I really feel as if this organization’s problems are my own.” The responses were given on a scale ranging from 1 (“strongly disagree”) to 5 (“strongly agree”). The scale’s reliability score (Cronbach’s alpha) was 0.91.

#### 3.2.4. CWB

A nine-item, abbreviated version of a scale by Bennett and Robinson (2000) was used. The example items are “Purposely wasted your employer’s materials/supplies” and “Said something obscene to someone at work to make them feel bad.” The scale’s reliability score (Cronbach’s alpha) was 0.88. This scale has been proven acceptable in the Chinese context and was also employed by Zhang et al. (2018) and Zhang et al. (2022) in the Chinese context. The reliability score (Cronbach’s alpha) for the turnover intention was 0.90.

#### 3.2.5. Control variables

We also included several theoretically relevant control variables. Tenure was controlled because scholars have proved that tenure is positively related to organizational affective commitment (Karen and Carlene, 2000). It was measured as the number of years an individual had been in the company (i.e., 1 = 1 year or less; 2 = 1–3 years; 3 = 3–5 years; 4 = 5–7 years; and 7 = 7 years or more). Age was also controlled for, as an individual’s age may influence their organizational affective commitment (Cohen, 1993). It was measured on a five-point scale with an interval of 10 years (i.e., 1 = 25 years old or younger; 2 = 25–35 years old; 3 = 35–45 years old; 4 = 45–55 years old; and 5 = older than 55 years). Gender was controlled because women have been found to have a higher organizational affective commitment (Aven et al., 1993). It was measured with a dichotomous variable (1 = male and 2 = female). Finally, we also controlled the education level because it is related to CWB (Wu et al., 2022).

### 3.3. Statistical analysis

Although this study used a multi-stage and multi-source method to collect data, there may still be some common methodological bias because subordinates evaluated two main variables. For this reason, based on the suggestions of Podsakoff et al. (2012), we employed Harman single-factor analysis using SPSS 21.0 to test potential common method bias. The result showed that 62.63% of the variance was explained by factors with eigenvalues greater than 1, and 37.74% of the variance was explained by the first factor.

Secondly, we conducted a series of confirmatory factor analyses (CFA) using Mplus 8.3 to demonstrate the construct validity of the major variables included in this study, namely turnover intention, organizational affective commitment, and CWB. The results of the measurement model indicated that the baseline model yielded a fit: *χ^2^* (df) = 2.02, *p* < 0.001, CFI = 0.91, TLI = 0.90, SRMR = 0.05, and RMSEA = 0.07. Furthermore, it was significant at the 0.001 level. Compared to the two-factor model [*χ^2^* (df) = 3.17, *p* < 0.001, CFI = 0.81, TLI = 0.78, SRMR = 0.08, RMSEA = 0.10] and the one-factor model [*χ^2^* (df) = 10.34, *p* < 0.001, CFI = 0.70, TLI = 0.66, SRMR = 0.08, RMSEA = 0.13], the baseline model was best suited to our data.

Moreover, we used Hierarchical ordinary least squares (OLS) regression analyses in SPSS 21.0 to test our hypotheses. Following the recommendation of Cohen et al. (2003), we mean-centered turnover intention and organizational affective commitment and calculated product terms to represent the interactions of turnover intention with employment type and organizational affective commitment. We also used bootstrapping methods to construct bias-corrected confidence intervals on the bias of 5,000 random samples to test the mediated moderation effect.

## 4. Results

### 4.1. Descriptive statistics and correlations

**Figure 2:**
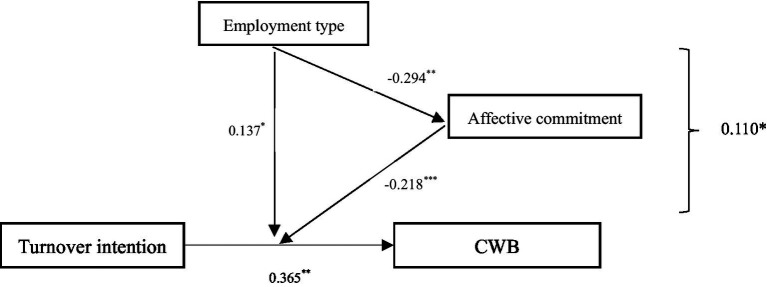
Model results. *p < 0.05; **p < 0.01; ***p < 0.001 (two tailed).

Table 1 displays the means, standard deviations, and correlations of the variables in this study. We conducted a multiple regression analysis to test all the hypotheses simultaneously; Table 2 reports the results of these analyses. Figure 2 presents the hypothesized effects.

**Table 2:** Hierarchical regression results.

	**AC**		**CWB**
	Model 1	Model 2	Model 3	Model 4	Model 5
Gender	0.041	0.114	0.102	0.053	0.106
Age	0.007	0.021	0.048	0.033	0.050
Education	0.072	−0.080^*^	−0.041	−0.072	−0.046
Tenure	−0.001	−0.055	−0.012	−0.042	−0.007
Type	−0.294^**^		0.303^***^		0.235^***^
TI		0.365^***^	0.141	0.358	0.314
AC				−0.121	−0.096
TI*type			0.137^*^		0.022
TI*AC				−0.252^***^	−0.218^***^
*R*^2^	0.08	0.391	0.438	0.483	0.489
*F*	3.586^**^	26.295^***^	24.347^***^	27.044^***^	23.340^***^

*N* = 211. TI: turnover intention; AC: affective commitment; CWB: Counterproductive work behavior.

*p < 0.05; **p < 0.01; ***p < 0.001 (two tailed).

### 4.2. Hypothesis test

Hypothesis 1 predicted that turnover intention would positively affect CWB. After entering the control variables, the results from regression analyses indicated that turnover intention was significantly related to CWB (as shown in Figure 2; Table 2, *β* = 0.365, *p* < 0.001), supporting Hypothesis 1.

**Figure 3:**
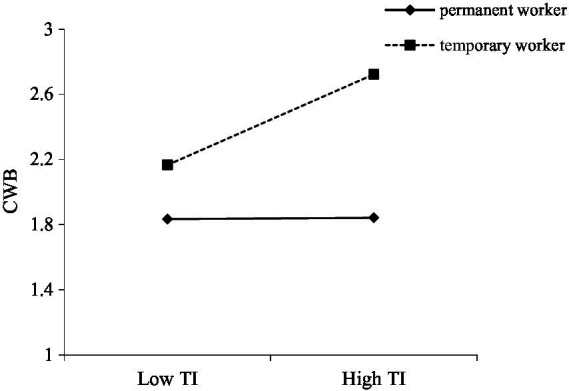
The moderating effect of employment type.

Hypothesis 2 predicted that employment type moderates the positive relationship between turnover intention and CWB. The results of the regression analyses supported the moderate effect of employment type (as shown in Figure 2; Table 2, *β* = 0.137, *p* < 0.05), supporting Hypothesis 2. Simple slopes suggested that turnover intention was more strongly related to CWB when the employees were temporary workers compared to when they were permanent workers (see Figure 3).

**Figure 4:**
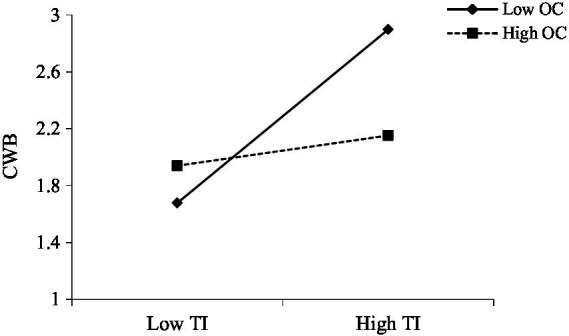
The moderating effect of organizational affective commitment.

We then tested Hypotheses 3a and 3b. In support of Hypothesis 3a (as shown in Figure 2; Table 2), the employment type was negatively associated with organizational affective commitment (*β* = −0.294, *p* < 0.01). In support of Hypothesis 3b, a moderated regression analysis showed that turnover intention and organizational affective commitment interacted with the predicted CWB of the individuals (*β* = −0.218, *p* < 0.001). Simple slopes showed that the form of the moderating effect of organizational affective commitment mirrored the moderating effect of employment type: the turnover intention was positively associated with CWB when individuals’ organizational affective commitment was high but not when it was low (see Figure 4).

We then examined whether organizational affective commitment mediated the moderating effect of employment type on the relationship between turnover intention and CWB. We began with the coefficients from the preceding analyses and utilized bootstrapping methods to construct bias-corrected confidence intervals that were based on 5,000 random samples with replacements from the full sample. The indirect effect from the full sample was 0.031. In accordance with Hypothesis 4, the 95% confidence interval from the bootstrap analysis excluded zero (0.003, 0.086). These results supported Hypothesis 4, showing that organizational affective commitment mediated the moderating effect of employment type on the association between turnover intention and CWB.

## 5. Discussion

Previous studies on turnover intention have mainly focused on exploring the predictors of turnover intention, and a few studies have investigated the impact of turnover intention on employees’ organizational citizenship behavior using cross-sectional data (Burris et al., 2008; Hom et al., 2012; Hancock et al., 2013). Considering the prevalence of flexible employment management practices and higher employee turnover rates, this study aimed to examine how individuals’ turnover intention affects CWB as well as whether and why there are differences in this association among permanent and temporary workers based on the psychological contract process perspective. Our results indicated that employees’ turnover intention is positively related to their subsequent CWB, and permanent workers are less likely to engage in CWB compared to temporary workers because of the former’s higher organizational affective commitment. Our result revealed that, compared to temporary workers, permanent workers’ higher organizational affective commitment enables them less likely to engage in CWB when they have turnover intention. This is because permanent workers’ higher organizational affective commitment enables employees to take actions based on the direction of achieving organizational goals, while CWB runs counter to organizational interests. Thus, permanent workers are unlikely to engage in CWB when they hold the turnover intention. As such, this study provides a more detailed exploration of the association between turnover intention and CWB, which could also contribute to the literature on turnover intention and the psychological contract theory.

### 5.1. Theoretical implications

The current research makes significant contributions to the literature. First, unlike previous studies, which mainly considered turnover intention as an organizational outcome (Hom et al., 2012; Park and Min, 2020), we have emphasized the impairing behavioral outcome of turnover intention. Previous studies on turnover intention have mainly focused on exploring the predictors of turnover intention, these studies have neglected the devastating impact of individuals’ turnover intention on CWB in the hybrid employment scenario. Moreover, our findings provide novel insights into the relative consequences of turnover intention on various groups of employees (i.e., temporary workers and permanent workers). Specifically, we confirmed the association between turnover intention and subsequent CWB in the context of Chinese diverse employment. Our study responds to recent calls to explore turnover intention more comprehensively (Cohen et al., 2016). Our research indicated that employees’ turnover intention is positively related to their subsequent CWB, which broadens the literature on turnover intention and hybrid employment.

Second, our findings provide a fresh perspective regarding the impact of different employment types on individuals’ psychology and behavior (Probst et al., 2018a,b; Wilkin et al., 2018; de Jong et al., 2019). While previous studies have examined the association between employment status and individuals’ behaviors (Boyce et al., 2007; De Cuyper et al., 2009; de Jong et al., 2019), they have not examined whether the association between turnover intention and CWB differs between temporary and permanent workers. To address this research gap, this study examined how the association between turnover intention and CWB differs across permanent and temporary workers. Our results demonstrated that temporary workers with turnover intention are more likely to engage in CWB than permanent workers. Thus, our study extends the influence of turnover intention by exploring different CWB responses between employees with turnover intention who are under different types of employment.

Third, this study introduces individuals’ organizational affective commitment to explain why such different associations occur in the flexible employment environment. Our results demonstrated that permanent workers are less likely to engage in CWB compared to temporary workers because of the former’s higher organizational affective commitment. Previous studies have suggested that temporary employees construct complicated psychological contracts with their organizations, which mainly consist of transactional psychological contracts and are supplemented by relational psychological contracts (De Cuyper et al., 2009). In contrast, permanent employees’ psychological contracts featured form relational psychological contracts (Saunders and Thornhill, 2006). Such different psychological contract contents enable employees to hold differing degrees of organizational commitment. Our results also indicate that permanent workers, who usually have higher organizational commitment, are less likely to exhibit CWB when they are faced with a psychological contract breach in the form of turnover intention. This finding is inconsistent with previous studies indicating that relational psychological contract violation exerts a more damaging influence on individuals’ behavior (Grimmer and Oddy, 2007). Our research shows that the emotional employee–organization relationship (organizational commitment/relational psychological contract) will buffer the effect of turnover intention on the CWB, making the linear relationship between the relational contract breach and CWB more complex, which increased the knowledge in this field. We have thus expanded psychological contract theory by identifying the moderated mediating role of organizational affective commitment.

Finally, our findings contribute to the psychological contract theory by extending it to the Chinese context and exploring how different content of psychological contracts in the form of employment status influence the impact of turnover intention on CWB. This study was conducted in the working environment of Asian organizations, responding to the call for more psychological contract research to be conducted in an Asian working environment by researchers (Kutaula et al., 2020). It is well known that China has a strong cultural context, and the workplace is no exception. Therefore, psychological contract research in the Chinese context is conducive to a deeper understanding of the employee–organization relationship from the Chinese perspective and the corresponding behavioral results.

### 5.2. Managerial implications

Our research offers important practical implications for organizations. First, Managers should not only focus on the impact of quitting on the organization caused by turnover intention, but also the other dark behaviors, such as CWB. Our results suggest that employees with turnover intention are then inclined to engage in CWB, thus having an impairing impact on organizational outcomes. Therefore, managers need to pay attention to those employees with turnover intention who are still staying in the organization. On one hand, organizational managers should further investigate what factors facilitate their turnover decision and adopt management practices that can help avoid employee turnover intention. For example, managers could put more effort into promoting job embeddedness and providing more organizational support (Hom and Kinicki, 2001) because prior studies have suggested that this practice could inhibit employees’ turnover intention. On the other hand, organizational managers should monitor those employees with turnover intention to avoid potential damage.

Second, in the flexible employment context, the proportion of quitting cognition has risen sharply, and managers need to realize that employees’ quitting cognition has different effects on their behavior across employment statuses. Our research shows that we need to pay more attention to the anti-productive behavior of short-term hired employees, as they are more exposed to planned resignations and are less psychologically attached to their organizations. While flexible employment helps organizations reduce labor costs and acquire external knowledge (De Stefano et al., 2019), such employment practices may also trigger potential risks (i.e., higher CWB). Managers need to be aware of these potential negative outcomes and evaluate the advantages and disadvantages of flexible employment.

Finally, our results suggest that organizational affective commitment plays an important mitigating role in the association between turnover intention and CWB. Therefore, managers need to balance the costs and benefits of investing in non-fixed-term employees. On one hand, organizations should try to cultivate employees’ organizational affective commitment by implementing human resource investment and other management practices. For example, research has shown that teamwork, perceived organizational support, and employee training contributes to improving individuals’ organizational affective commitment (Hanaysha, 2016; Kim et al., 2016). On the other hand, whether an attempt to establish a committed relationship with contract workers is worthwhile depends on the specific situation. After all, the impact of employees’ CWB on the organization is different in work scenarios.

### 5.3. Limitations and future directions

While this study makes several important contributions to the extant literature, it also has several limitations. First, our research only explores the association between turnover intention and the CWB of temporary and permanent employees. However, other forms of nonstandard employment have emerged in organizations, such as self-employed workers and gig workers (Cropanzano et al., 2022). Future research could examine the behavior differences between these nonstandard and permanent workers based on different perspectives. For example, it could investigate the different coping strategies of permanent and nonstandard employees responding to customer mistreatment.

Moreover, our research only emphasized the impact of turnover intention on employees’ CWB and did not explore other behavioral results in multiple employment situations. Future research can explore the impact of turnover intention on other behavioral performances of employees, such as task performance and or prosocial behavior. Moreover, more specific negative behaviors related to turnover intention could be further explored, such as whether withdrawal behavior or unethical behavior. It is believed such research in more detail on the behavioral consequences of turnover intention will add more knowledge to this field and bring inspiration to practice.

Third, our research explores the role of turnover intention and employment types interactively in predicting the role of CWB, but the ways to mitigate the negative effects of temporary employment on the association between turnover intention and CWB have not been explored. Our research raises important unanswered questions about the boundary conditions for the moderating effects of employment types. Future research can further explore other possible interventions that can be implemented to eliminate the negative effects of flexible employment. Such further investigation could contribute to the literature on flexible employment.

## 6. Conclusion

Our research sheds new light on why employees with turnover intention are involved in the CWB in multiple employment situations. Previous studies have primarily focused on exploring the antecedent variable of turnover intention. However, employee turnover intention is becoming common with the popularity of flexible employment related to the loose employee-organization relationship. Thus, we strive to investigate the employee’s behaviors when he or she is still working in the organization while with turnover intention. Our results show that employees’ turnover intention is positively related to their subsequent CWB. We also strive to explore whether and how the association between turnover intention and CWB is different across temporary workers and permanent workers. The results indicate that the impact of turnover intention on CWB is stronger for temporary workers than permanent ones because of their lower organizational commitment. This finding demonstrated that permanent workers’ higher organizational affective commitment makes them less likely to engage in CWB when they have higher turnover intention. This research expands the limited scholarly inquiry into behavior results of turnover intention (Burris et al., 2008; Hom et al., 2012) and provides insights into the management of employees’ CWB in the context of hybrid employment.

## Data availability statement

The original contributions presented in the study are included in the article/supplementary material, further inquiries can be directed to the corresponding author.

## Author contributions

XL is in charge of research design, paper writing, and data analysis. WL is also responsible for research design, paper writing, and data collecting. SL put forward the innovation points of the paper and gave corresponding suggestions on the paper’s research design. CQ was also responsible for original paper writing and data analysis. All authors contributed to the article and approved the submitted version.

## Funding

This research was funded by the National nature science Foundation of Youth, Grant number, 72002072; the Humanities and Social Sciences Youth Project of the Ministry of Education, Grant number, 19YJC630106; National nature science Foundation of Youth, Grant number, 71902037; National Natural Science Foundation of Key, Grant number, 71832003; Philosophy and Social Science Project of Guangdong Province, Grant number, GD20YGL01; The Yangcheng Young Scholars Project of 13th Five Year Plan of Guangzhou City Philosophy and Social Sciences, Grant number, 2020GZQN04.

## Conflict of interest

The authors declare that the research was conducted in the absence of any commercial or financial relationships that could be construed as a potential conflict of interest.

## Publisher’s note

All claims expressed in this article are solely those of the authors and do not necessarily represent those of their affiliated organizations, or those of the publisher, the editors and the reviewers. Any product that may be evaluated in this article, or claim that may be made by its manufacturer, is not guaranteed or endorsed by the publisher.
